# High Q Resonant Graphene Absorber with Lossless Phase Change Material Sb_2_S_3_

**DOI:** 10.3390/nano11112820

**Published:** 2021-10-24

**Authors:** Qi Meng, Xingqiao Chen, Wei Xu, Zhihong Zhu, Shiqiao Qin, Jianfa Zhang, Xiaodong Yuan

**Affiliations:** 1 College of Advanced Interdisciplinary Studies, National University of Defense Technology, Changsha 410073, China; Monky19@163.com (Q.M.); chenxingqiao14@nudt.edu.cn (X.C.); weixu08a@163.com (W.X.); zzhwcx@163.com (Z.Z.); sqqin8@nudt.edu.cn (S.Q.); x.d.yuan@163.com (X.Y.); 2 Hunan Provincial Key Laboratory of Novel Nano-Optoelectronic Information Materials and Devices, National University of Defense Technology, Changsha 410073, China

**Keywords:** graphene absorbers, phase change material, high Q

## Abstract

Graphene absorbers have attracted lots of interest in recent years. They provide huge potential for applications such as photodetectors, modulators, and thermal emitters. In this letter, we design a high-quality (Q) factor resonant graphene absorber based on the phase change material Sb_2_S_3_. In the proposed structure, a refractive index grating is formed at the subwavelength scale due to the periodical distributions of amorphous and crystalline states, and the structure is intrinsically flat. The numerical simulation shows that nearly 100% absorption can be achieved at the wavelength of 1550 nm, and the Q factor is more than hundreds due to the loss-less value of Sb_2_S_3_ in the near-infrared region. The absorption spectra can be engineered by changing the crystallization fraction of the Sb_2_S_3_ as well as by varying the duty cycle of the grating, which can be employed not only to switch the resonant wavelength but also to achieve resonances with higher Q factors. This provides a promising method for realizing integrated graphene optoelectronic devices with the desired functionalities.

## 1. Introduction

Graphene absorbers have attracted lots of interest in recent years [1,2,3]. They provide huge potential for applications such as photodetectors [4], modulators [5,6,7,8], and thermal emitters [9,10]. Meanwhile, high-Q planar resonator structures have been extensively studied in the past few years. Various structures have been studied to achieve high Q resonances, including photonic crystals [11,12,13], dielectric metasurfaces [14,15], nanocavities [16,17] and guided mode resonance structures [18,19,20] (GMRs). Such types of high Q photonic devices provide a promising future for novel photonic and optoelectronic devices, such as thermal emitters [21,22], ultrathin mirrors [23,24], ultrafast photodetectors [25,26], polarization devices [27,28] and nonlinear optics [20,29]. Thus, the realization of high Q resonant graphene absorbers is of particular importance for many applications.

Phase change materials (PCM), such as ${\mathrm{Ge}}_{2}{\mathrm{Sb}}_{2}{\mathrm{Te}}_{2}$(GST) and ${\mathrm{VO}}_{2}$, have has been used in optically tunable nanophotonic devices for a long time [30,31,32], such as thermal camouflage [33], dynamic color display [34,35,36], beam steering [37], and light modulation [38,39,40,41]. Previous work has been able to write, erase, and rewrite two-dimensional binary or gray-scale functional patterns into nano-scale GST films through customized lasers, to induce local refractive index changes and construct nanophotonic devices [42,43]. Electrode heating can also be applied to write and erase the patterns in GST, achieving a polarization-insensitive phase gradient metasurface [44]. However, the inherent absorption of most previous phase change materials, such as GST and ${\mathrm{VO}}_{2}$, in the near-infrared and visible light bands limits their resonant Q factors and, thus, the performances in many photonic devices.

In this study, we propose a high Q rewriteable resonant graphene absorber with the phase change material antimony trisulphide (${\mathrm{Sb}}_{2}S_{3}$).The absorption of ${\mathrm{Sb}}_{2}S_{3}$ in the near-infrared is zero, which promises a relatively high Q factor for the absorber. We can construct refractive index grating in the ${\mathrm{Sb}}_{2}S_{3}$ layer by local crystallization (i.e., with laser or ion beam), which greatly simplifies the processing of photonic devices. We can also easily “write” or “erase” the grating layer to adjust the structural parameters of photonic devices. Numerical simulations indicate that the graphene integrated ${\mathrm{Sb}}_{2}S_{3}$ absorber achieves nearly 100% absorption at 1547.9 nm, with the spectral linewidths (full width at half maximum, FWHM) of 2.4 nm, corresponding a high Q factor of 644. We can engineer the absorption spectrum by adjusting the duty cycle or crystalline fraction, to tune the resonant wavelength or obtain a much higher Q factor.

## 2. Result and Discussion

**Figure 1:**
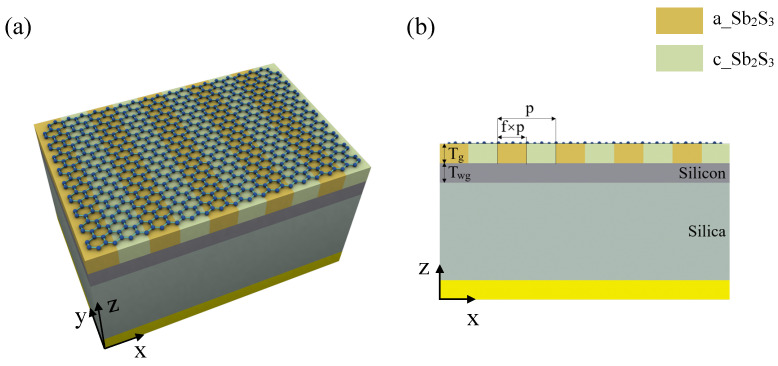
Schematic of the graphene absorber with ${\mathrm{Sb}}_{2}S_{3}$. (**a**) Schematic of graphene absorber with ${\mathrm{Sb}}_{2}S_{3}$. From top to bottom, is the graphene, ${\mathrm{Sb}}_{2}S_{3}$ grating, a silicon waveguide layer, a silica substrate layer, and an Au mirror. The structure is intrinsically flat and no etching process is needed. (**b**) Sideview of the proposed absorber.

Local heating, laser illumination, or ion beam irradiation can be used to induce the phase transition of the ${\mathrm{Sb}}_{2}S_{3}$ film, thus make a grating layer with crystalline ${\mathrm{Sb}}_{2}S_{3}$ ($c\text{\_}{\mathrm{Sb}}_{2}S_{3}$) and amorphous ${\mathrm{Sb}}_{2}S_{3}$ ($a\text{\_}{\mathrm{Sb}}_{2}S_{3}$). Here, it should be indicated that the graphene can survive under the light intensities of tens of thousands GW/m${}^{2}$ [45], and the pulse intensity required to accomplish transition is about 10 GW/m${}^{2}$ [46], which is much lower than that of destroying a monolayer graphene. Moreover, for the ion beam, the graphene can be transferred to the top of the structure after the grating is produced to avoid damage. Figure 1a is the schematic of the integrated ${\mathrm{Sb}}_{2}S_{3}$ absorber. From top to bottom, is the monolayer graphene, ${\mathrm{Sb}}_{2}S_{3}$ grating, a silicon (Si) waveguide layer, a silica (${\mathrm{SiO}}_{2}$) substrate layer and an Au mirror, with the thickness of graphene, ${\mathrm{Sb}}_{2}S_{3}$, Si, ${\mathrm{SiO}}_{2}$ and Au is 0.34 nm, 40 nm, 205 nm, 380 nm and 150 nm, respectively. The sideview of the ${\mathrm{Sb}}_{2}S_{3}$ absorber is shown in Figure 1b, and the period of the absorber is *p* = 550 nm, and width of $c\text{\_}{\mathrm{Sb}}_{2}S_{3}$ is w=f×p, where *f* = 0.5 is the duty cycle of the absorber.

The numerical simulations are conducted in a fully three-dimensional finite element technology (in COMSOL Multiphysics). Since our structure is assumed to be “infinitely extending” in the y direction, we use a two-dimensional model for simulations (x–z plane). We used Floquet periodic boundary conditions in the x-direction, and port boundary conditions at the top and bottom of the model along the z-direction. In the simulation, ${\mathrm{SiO}}_{2}$ and Si are regarded as a lossless medium in the near-infrared, with a refractive index of $n_{{\mathrm{SiO}}_{2}}=1.45$ and $n_{\mathrm{Si}}=3.42$. The permittivity of gold was described by the Drude model with plasma frequency $\omega_{p}=1.37\times 10^{-16}$ s${}^{-1}$ and the damping constant $\omega_{t}=12.15\times 10^{-13}$ s${}^{-1}$, which was three times larger than the bulk value. The complex refractive indices of $c\_{\mathrm{Sb}}_{2}S_{3}$$n_{c}$ and $a\text{\_}{\mathrm{Sb}}_{2}S_{3}$$n_{a}$ are taken from experimental measurements [46], and the absorption of ${\mathrm{Sb}}_{2}S_{3}$ is zero in the near-infrared region. For example, the complex refractive indices of $c\text{\_}{\mathrm{Sb}}_{2}S_{3}$ and $a\text{\_}{\mathrm{Sb}}_{2}S_{3}$ at the wavelength of 1550 nm are $n_{c}$ = 3.308 + 0i and $n_{a}$ = 2.712 + 0i, respectively. Furthermore, Graphene is defined by a conductivity model [47]:

(1) $$\begin{array}{cc} \sigma_{\omega } & =\sigma_{\mathrm{intra}}\left(\omega \right)+\sigma_{\mathrm{inter}}\left(\omega \right)\\ \sigma_{\mathrm{intra}}\left(\omega \right) & =\frac{2e^{2}k_{B}T}{\pi \hbar^{2}}\frac{i}{\omega +i\tau^{-1}}\ln\left[\left.2\cos h(\frac{E_{f}}{2k_{B}T}\right)\right]\\ \sigma_{\mathrm{inter}}\left(\omega \right) & =\frac{e^{2}}{4\hbar }\left[\left.\frac{1}{2}+\frac{1}{\pi }\arctan(\frac{\hbar \omega -2E_{f}}{2k_{B}T}\right)-\frac{i}{2\pi }\ln\frac{\left(\hbar \omega +2E_{f}{)}^{2}\right.}{\left(\hbar \omega -2E_{f}{)}^{2}+4\left(k_{B}{T)}^{2}\right.\right.}\right] \end{array}$$

where *ℏ* is the reduced Planck constant, $k_{B}$ is the Boltzmann constant. Here, we have $E_{f}∼0ev$, $\tau =10^{-14}$ s, and T=300 K for the Fermi level, relaxation time, and temperature, respectively. The relation between surface conductivity and permittivity of graphene is:

(2) $$\begin{array}{c} \varepsilon_{r}=1+i\frac{\sigma }{\varepsilon_{0}\omega d} \end{array}$$

Here, σ is the surface conductivity and *d* is the thickness of graphene. For the graphene monolayer, we have d=0.34 nm, ω is the angular frequency, and $\varepsilon_{0}$ is the vacuum permittivity, respectively.

**Figure 2:**
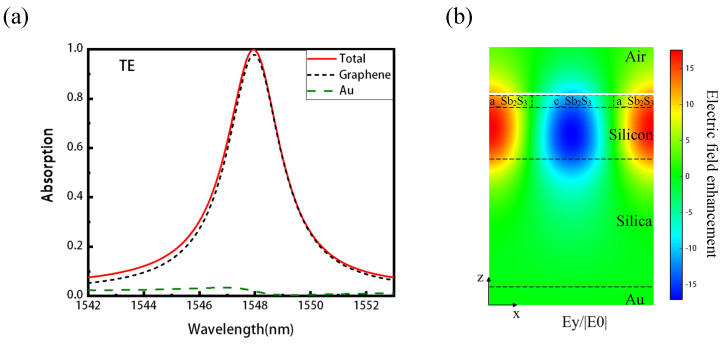
The simulation results of the proposed graphene absorber. (**a**) Absorption spectra for TE (y-polarized) polarization. The structure has near 100% absorption at 1547.9 nm, and the absorption of graphene reaches 99%. (**b**) The distributions of the normalized electric field in the y-direction at the resonance wavelength of 1547.9 nm.

Figure 2a shows the simulated absorption spectra of the graphene-integrated ${\mathrm{Sb}}_{2}S_{3}$ absorber. For transverse electric (TE) polarized light at normal incidence, a typical high Q guided mode resonance is excited around the wavelength of 1547.9 nm, with a resonant absorption of nearly 100% at the peak (the transverse magnetic (TM) mode can also excite the resonances in the studied structure, but at different wavelengths). An integration of resistive losses in the numerical simulations show that only an ignorable part of the light is absorbed by Au (the green dotted lined), while over 99% of the light is absorbed by the graphene (the black dotted lined). Owing to the existence of the ${\mathrm{SiO}}_{2}$ substrate layer, the interplay of the GMR resonance of the ${\mathrm{Sb}}_{2}S_{3}$ and the Fabry-Pérot (F-P) effect on the cavity greatly enhances the absorption of the graphene [20]. Figure 2b is the distributions of the electric field in the y-direction at the resonance wavelength of 1547.9 nm, which means that most of the energy is trapped in the waveguide.

**Figure 3:**
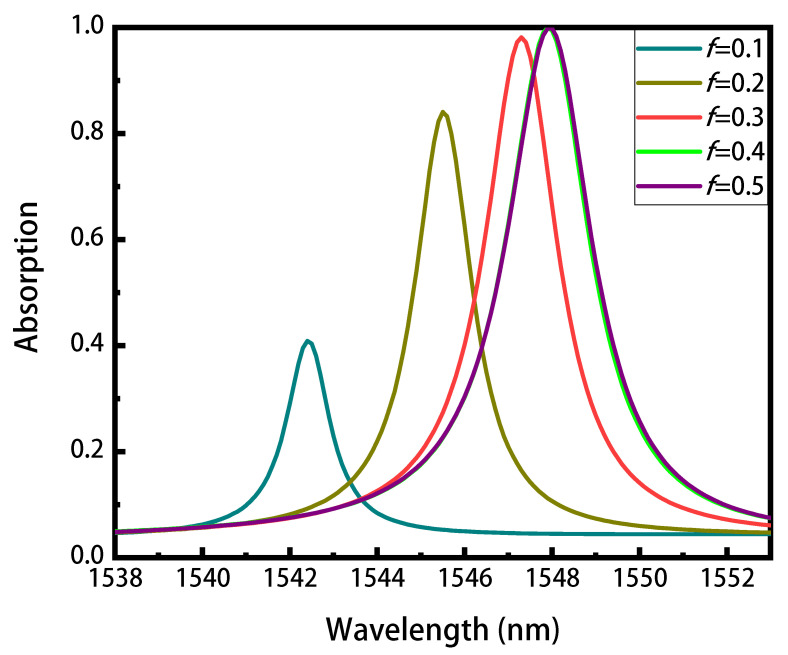
The absorption spectra of the graphene absorber with different duty cycles. As the duty cycle decreases from 0.5 to 0.1, the peak absorption and the linewidth decrease.

The excitation of the guided mode resonance can be controlled by changing the geometric parameters of the structure (such as period and duty cycle), as mentioned above. We now fix the period of the grating, and tune the absorption spectra by changing the duty cycle. As shown in the Figure 3, as the duty cycle decreases from *f* = 0.5 to *f* = 0.1, the resonance wavelength shifts from 1547.9 nm to 1542.4 nm and the maximum resonant absorption decreases from 99.83% to 40.88%. At the same time, the spectral linewidths (FWHM) decrease from 2.4 nm to 1.4 nm, corresponding to an increase of the Q factor from 644 to 1101 (Q = $\lambda_{r}$/FWHM, $\lambda_{r}$ is the resonant wavelength). Such phenomena can be explained by the concept of critical coupling derived from the coupled mode theory [48], that is, when the mode leakage rate γ is equal to the absorption rate α of the structure, the perfect absorption occurs. In this work, the perfect absorber is designed by integrating graphene with a resonator illuminated from one side, where a back mirror is used to completely block the transmission. Here, the mode leakage rate denotes the energy coupled in the structure, while the absorption rate α is determined by the graphene absorption coefficient (a constant) and the field intensity in the graphene layer. The decrease of duty cycle reduces the effective refractive index of the ${\mathrm{Sb}}_{2}S_{3}$ grating layer, thus introducing the blueshift and the decrease of the leakage rate γ. As the leakage rate γ is no longer equal to the absorption rate α, the absorption peak decreases for the mismatch of the critical coupling. Previous work has indicated that the linewidth (FWHM) is proportional to (γ + α) [48], the FWHM therefore decreases as that of the leakage rate γ.

**Figure 4:**
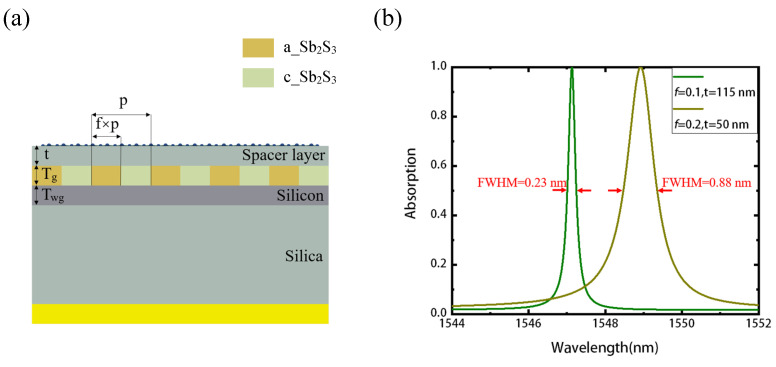
Engineered perfect absorption with high Q and narrow band. (**a**) Schematic of the absorber with a ${\mathrm{SiO}}_{2}$ spacer layer between graphene and the phase change layer. As the duty cycle varies, one can change the thickness of the spacer layer to meet the critical coupling condition and, thus, realize perfect absorption. (**b**) The structure achieves nearly 100% absorption again for the duty cycle *f* = 0.1 and *f* = 0.2 with 115 nm ${\mathrm{SiO}}_{2}$ and 50 nm ${\mathrm{SiO}}_{2}$, respectively.

This type of mismatch results from the decrease of the mode leakage rate γ as the duty cycle *f* is reducing, and it can be avoided so long as the effective absorption rate of graphene α decreases, where the critical coupling condition meets again. As shown in the Figure 4a, we now add a spacer (coating) layer ${\mathrm{SiO}}_{2}$ between the ${\mathrm{Sb}}_{2}S_{3}$ grating and graphene, thus moving the graphene away from the waveguide layer where most of the enhanced electric field is localized. As the effective absorption rate of graphene decreases, we can achieve critical coupling and perfect absorption for smaller duty cycles. Taking duty cycle *f* = 0.1 and *f* = 0.2 as the example, as shown in the Figure 4b, with the thickness of the spacer layer t = 115 nm and t = 50 nm, respectively; thus, the absorber structure achieves perfect absorption in both cases. The linewidth (FWHM) of *f* = 0.1 and *f* = 0.2 are 0.23 nm and 0.88 nm, corresponding to high Q of 6726 and 1760, respectively, which is the consequence of the decrease of the mode leakage rate γ and absorption rate α.

**Figure 5:**
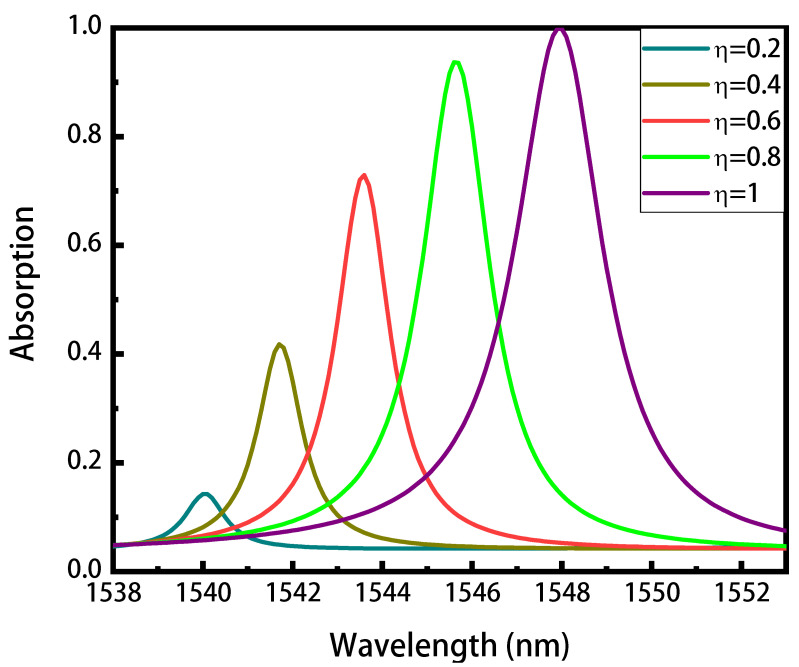
The absorption spectra of the graphene absorber with different crystalline fractions of the ${\mathrm{Sb}}_{2}S_{3}$ grating (with duty cycle fixed at *f* = 0.5).

A previous work has demonstrated that there is an intermediate state of ${\mathrm{Sb}}_{2}S_{3}$ between $a\text{\_}{\mathrm{Sb}}_{2}S_{3}$ and $c\text{\_}{\mathrm{Sb}}_{2}S_{3}$, and has been able to use different power femtosecond laser pulses to control the crystallization fraction of ${\mathrm{Sb}}_{2}S_{3}$ [35]. The effective dielectric constant of $c\text{\_}{\mathrm{Sb}}_{2}S_{3}$ can be obtained by the Lorenz-Lorentz relationship along with the Bruggeman mix rule [49]:

(3) $$\begin{array}{c} \frac{\varepsilon_{eff}\left(\lambda \right)-1}{\varepsilon_{eff}\left(\lambda \right)+2}=\eta \times \frac{\varepsilon_{c\text{\_}Sb_{2}S_{3}}\left(\lambda \right)-1}{\varepsilon_{c\text{\_}Sb_{2}S_{3}}\left(\lambda \right)+2}+\left(1-\eta \right)\times \frac{\varepsilon_{a\text{\_}Sb_{2}S_{3}}\left(\lambda \right)-1}{\varepsilon_{a\text{\_}Sb_{2}S_{3}}\left(\lambda \right)+2} \end{array}$$

where $\varepsilon_{c\text{\_}Sb_{2}S_{3}}\left(\lambda \right)$ and $\varepsilon_{a\text{\_}Sb_{2}S_{3}}\left(\lambda \right)$ are the wavelength-dependent permittivity of crystalline and amorphous ${\mathrm{Sb}}_{2}S_{3}$, respectively, and η is the crystalline fraction of $c\text{\_}{\mathrm{Sb}}_{2}S_{3}$. Now, we fixed the duty cycle *f* = 0.5 and vary the crystallization fraction of $c\text{\_}{\mathrm{Sb}}_{2}S_{3}$. The absorption spectra with the different crystallization fractions are shown in Figure 5. The decrease of the crystallization fraction causes a similar influence on the absorption as the decrease of the duty ratio, where the resonance wavelength shifts from 1547.9 nm to 1540.0 nm and the maximum resonant absorption decreases from 99.83% to 14.25%. Furthermore, the spectral linewidths (FWHM) decrease from 2.4 nm to 1.6 nm, corresponding to an increase of Q factor from 644 to 962.

**Figure 6:**
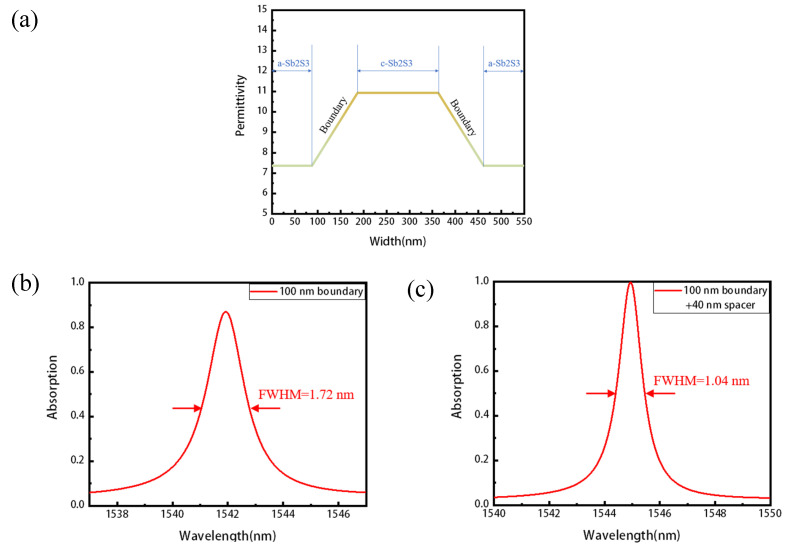
Considering the influence of the boundary between amorphous and crystalline ${\mathrm{Sb}}_{2}S_{3}$. (**a**) The linear graded index with a width of 100 nm at each boundary between amorphous and crystalline ${\mathrm{Sb}}_{2}S_{3}$. (**b**) The changes of absorption and Q after considering graded index boundary. (**c**) Achieving perfect absorption again after adding a spacer layer.

To investigate the influence of the boundary between amorphous and crystalline PCM, we also set a linear graded-index with the width of 100 nm at each boundary between amorphous and crystalline ${\mathrm{Sb}}_{2}S_{3}$, with other parameters kept the same as that of Figure 2 (see the Figure 6a). As is shown in Figure 6b, a similar sharp resonance is observed near the telecom wavelength range. The absorption decreases to 87%, while FWHM decreases from 2.40 nm to 1.72 nm, with a Q factor of 896. Those changes can be easily understood, as a linear graded-index boundary means a decrease of the refractive index contrast and the mode leakage rate γ. To achieve perfect absorption again, we can add a spacer layer as that of Figure 4 to reduce the absorption rate α. After adding a 40 nm spacer layer, the resonant grating absorber achieves nearly 100% absorption again, with FWHM = 1.04 nm, corresponding to a much higher Q factor of 1485 (see the Figure 6c).

**Figure 7:**
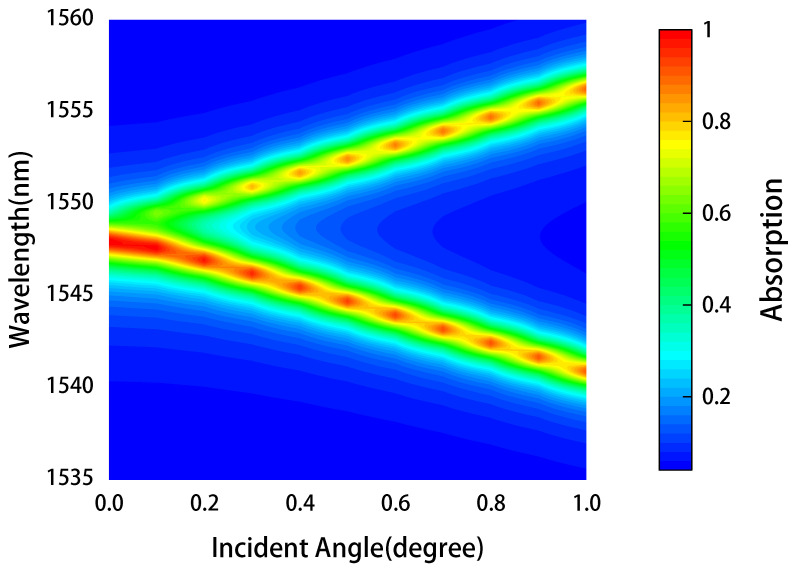
Angular dependence of the graphene absorber structure for TE polarization, the parameters of the structure are the same as those of Figure 2. And the calculated angle ranges from 0 to 1 degree.

Figure 7 illustrates the angular dependence of the graphene absorber (the parameters kept the same as that of Figure 2). Different from the metamaterials or plasmonic absorbers with localized resonances, whose angular dependence is generally weak. The guide mode resonant (GMR) grating-based absorber here is sensitive to the incident angle, as shown in the figure, with the incident angle increased from 0 to 1 degree, the single absorption peak split into two, and therefore, the greater the angle, the greater the distance between them. This angle-dependence comes from phase-matching and has been well studied [50]. It can be applied to directional thermal emitting [51], optical filtering [52], angle sensorinfg [53] and other applications.

## 3. Conclusions

In all, we have proposed a type of subwavelength resonant graphene absorber based on the phase change material ${\mathrm{Sb}}_{2}S_{3}$. The proposed structure is intrinsically flat, which is different from most previously demonstrated graphene absorbers in the near-infrared range. We can deposit the phase change material ${\mathrm{Sb}}_{2}S_{3}$ on the Si and then use customized laser pulses to realize the required structure (e.g., the grating structure). Other methods, such as electrothermal switching [54,55], could also be applied to induce the crystallization and amorphization of phase change materials. The ultralow loss of ${\mathrm{Sb}}_{2}S_{3}$ enables us to realize high Q factors up to thousands in the proposed structure. To achieve the high Q resonances, we have employed a classical guided-mode resonant grating structure, which generally consists of a grating layer, a waveguide layer with a relatively high refractive index, and a substrate. Here, the PCM with periodical distributions of amorphous and crystalline states works as a refractive index grating. Silica is the most widely used material in a semiconductor and is also widely used in photonics. It has a high refractive index and low loss at the studied telecom wavelength range. Thus, a Si layer is chosen as the waveguide layer. ${\mathrm{SiO}}_{2}$ with a relatively low refractive index and low loss was chosen as the substrate, and a reflective gold layer is used to block the transmission for perfect absorption. The thickness of the PCM, Si, and ${\mathrm{SiO}}_{2}$ has been optimized to realize the high-Q resonances and satisfy the critical coupling conditions for perfect absorption. Simulation results demonstrate that the absorber achieves nearly 100% absorption at 1547.9 nm, with linewidths of 2.4 nm, corresponding to a high Q factor of 644. Besides, we can continuously tune the absorption spectra not only by adjusting the duty cycle but also by changing the crystalline fraction. Resonant perfect absorption with a much higher Q factor up to 6726 is numerically demonstrated with a smaller duty cycle *f* = 0.1 and a spacing layer between the phase change material-based grating and graphene to reduce both the coupling rate and the absorption rate. This type of high Q resonant of two-dimension materials of the integrated phase change metadevice can also be applied to other atomical materials, such as ${\mathrm{MoS}}_{2}$ and ${\mathrm{WS}}_{2}$, whose absorption at visible and near-infrared wavelengths is considerable [56]. This provides a promising method for realizing integrated graphene and other two-dimensional material optoelectronic devices with desired functionalities such as photodetection, light generation, spatial light modulation, and others [57].

## Data Availability

The data presented in this study are available on request from the corresponding author.
